# Novel histone deacetylase inhibitor AR-42 exhibits antitumor activity in pancreatic cancer cells by affecting multiple biochemical pathways

**DOI:** 10.1371/journal.pone.0183368

**Published:** 2017-08-22

**Authors:** Yi-Jin Chen, Wen-Hung Wang, Wan-Yu Wu, Chia-Chi Hsu, Ling-Rung Wei, Sheng-Fan Wang, Ya-Wen Hsu, Chih-Chuang Liaw, Wan-Chi Tsai

**Affiliations:** 1 Department of Medical Laboratory Science and Biotechnology, Kaohsiung Medical University, Kaohsiung, Taiwan; 2 Department of Otolaryngology, Cathay General Hospital, Taipei City, Taiwan; 3 Department of Otolaryngology, Sijhih Cathay General Hospital, New Taipei City, Taiwan; 4 School of Medicine, Fu-Jen Catholic University, New Taipei City, Taiwan; 5 Center for Infectious Disease and Cancer Research, Kaohsiung Medical University, Kaohsiung, Taiwan; 6 Department of Hospital and Health Care Administration, Chia Nan University of Pharmacy & Science, Tainan, Taiwan; 7 Doctoral Degree Program of Marine Biotechnology, National Sun Yat-Sen University, Kaohsiung, Taiwan; 8 Department of Marine Biotechnology and Resources, National Sun Yat-sen University, Kaohsiung, Taiwan; 9 Department of Laboratory Medicine, Kaohsiung Medical University Hospital, Kaohsiung, Taiwan; University of South Alabama Mitchell Cancer Institute, UNITED STATES

## Abstract

**Objective:**

Pancreatic cancer is one of the most lethal types of cancer with a 5-year survival rate of ~5%. Histone deacetylases (HDACs) participate in many cellular processes, including carcinogenesis, and pharmacological inhibition of HDACs has emerged as a potential therapeutic strategy. In this study, we explored antitumor activity of the novel HDAC inhibitor AR-42 in pancreatic cancer.

**Methods:**

Human pancreatic cancer cell lines BxPC-3 and PANC-1 were used in this study. Real-time PCR, RT-PCR, and western blotting were employed to investigate expression of specific genes and proteins, respectively. Translocation of apoptosis-inducing factor was investigated by immunofluorescence and subcellular fractionation. The number of apoptotic cells, cell cycle stages, and reactive oxygen species (ROS) generation levels were determined by flow cytometry. Cell invasiveness was examined by the Matrigel invasion assay. Efficacy of AR-42 *in vivo* was evaluated by utilizing BxPC-3 xenograft mouse model.

**Results:**

AR-42 inhibited pancreatic cancer cell proliferation by causing G2/M cell cycle arrest via regulating expression levels of genes and proteins involved in cell cycle. AR-42 also induced ROS generation and DNA damage, triggering apoptosis of pancreatic cancer cells via both caspase-3-dependent and caspase-3-independent pathways. In addition, AR-42 increased expression levels of negative regulators of p53 (miR-125b, miR-30d, and miR33), which could contribute to lower expression level of mutant p53 in pancreatic cancer cells. Cell invasion assay showed that AR-42 reduced cancer cell aggressiveness and significantly diminished BxPC-3 xenograft tumor growth *in vivo*.

**Conclusion:**

AR-42, a novel HDAC inhibitor, inhibited pancreatic cancer cells by regulating p53 expression, inducing cell cycle arrest, particularly at the G2/M stage, and activating multiple apoptosis pathways. Additionally, AR-42 inhibited cell invasiveness and potently suppressed pancreatic cancer tumors *in vivo*. We conclude that by virtue of its multiple mechanisms of action, AR-42 possesses a considerable potential as an antitumor agent in pancreatic cancer.

## Introduction

Pancreatic cancer is the third leading cause of cancer-related deaths in the United States [1]. Although surgery remains the best way to treat localized disease, less than 20% of patients have operable pancreatic cancer at diagnosis. Moreover, 80% of patients with localized pancreatic cancer experience recurrence within three years after surgery [2]. Chemotherapy remains the preferred treatment modality for patients with locally advanced or metastatic pancreatic cancer, but a combination of poor response rate and short progression-free interval time results in a five-year survival rate of less than 5% [3]. Therefore, novel and effective therapeutic avenues are urgently needed for pancreatic cancer treatment.

Histone acetylation is an important determinant of gene expression. Acetylation is generally associated with elevated transcription, whereas deacetylated histones are often associated with gene repression. Histone deacetylases (HDACs) are enzymes that determine acetylation status of histones, thereby affecting chromatin structure and regulating the expression and activity of numerous proteins involved in both cancer initiation and cancer progression [4–6]. Pharmacological inhibition of HDACs has emerged as a potential therapeutic tactics in many types of cancer [7–10], and HDAC inhibitors (HDACis) have been used in clinical trials to treat a variety of malignancies [11–13]. HDACis can block cell proliferation, promote differentiation, and induce apoptosis, *i*.*e*., phenomena that impede growth of cancer cells.

AR-42, a derivative of hydroxamate-tethered phenylbutyrate, inhibits HDACs with a low nanomolar IC_50_ and is currently in Phase I/Ib trials for hematological malignancies and solid tumors [14]. AR-42 demonstrated anticancer activity in many cancers, including acute myeloid leukemia [15], multiple myeloma [16], prostate cancer [17], ovarian cancer [18], human glioma cells [19], and bladder cancer [20].

In this study, we evaluated antitumor effects of AR-42 in pancreatic cancer cells. Our data indicated that AR-42 exerted potent anticancer effects at submicromolar concentrations by inducing cell cycle arrest, stimulating apoptosis, and regulating expression of several miRNAs. Furthermore, we demonstrated that AR-42 suppressed pancreatic cancer growth in mouse subcutaneous tumor xenograft model. Taken together, our findings support further development of AR-42 for a clinical application in pancreatic cancer.

## Materials and methods

### Cell culture

BxPC-3 and PANC-1 (derived from primary tumor [21]) human pancreatic cancer cell lines were purchased from the American Type Culture Collection (ATCC; Manassas, VA) and cultured in RPMI-1640 medium supplemented with 10% fetal bovine serum (FBS) (Gibco, Grand Island, NY), 1% sodium pyruvate, and 1% penicillin/streptomycin in the atmosphere of 95% air and 5% CO_2_ at 37°C.

### Reagents

AR-42 was a kind gift from Arno Therapeutics, Inc. (Flemington, NJ). Stock solution of AR-42 (100 mM) was prepared in dimethyl sulfoxide (DMSO) (Sigma-Aldrich, St. Louis, MO) and stored at –20°C. For *in vivo* experiments, AR-42 was prepared as a suspension in a vehicle [10% DMSO, 0.5% methylcellulose (w/v), and 0.1% Tween 80 (v/v) in sterile water] for oral administration to xenograft-bearing athymic nude mice. Anti-cyclin B1 (GNS1), anti-cyclin B2 (H-105), anti-γH_2_AX, anti-survivin (D-8), anti-XIAP (A-7), anti-caspase 8, anti-apoptosis inducing factor (AIF) (E1), anti-mouse IgG-CFL 488, anti-p21 (C19), anti-E-cadherin (H108) and anti-p53 (DO-1) antibodies were purchased from Santa Cruz Biotechnology (Santa Cruz, CA) Anti-caspase 9 (C9), anti-caspase 3, and anti-PARP antibodies were from Cell Signaling Technologies (Beverly, MA). Anti-histone H4 antibody was purchased from Active Motif (Rixensart, Belgium). Anti-N-cadherin was from Genetex (GTX112733, GeneTex Inc., San Antonio, TX).

### Cell viability assay

Cell viability was assessed by the 3-(4,5-dimethylthiazol-2-yl)-2,5-diphenyltetrazolium bromide (MTT) assay. Briefly, BxPC-3 and PANC-1 cells (5 × 10^3^ cells per well) were seeded in 96-well plates and treated with test agents at various concentrations for fixed time intervals. To quantify cell viability, medium was replaced with 150 μL of fresh medium containing 10% MTT solution (Sigma-Aldrich). After incubation at 37°C for 1 h, MTT-containing solution was removed, and formazan crystals within cells were solubilized with 100 μL DMSO. Absorbance levels for each sample were measured at 595 nm by a microplate spectrophotometer (Bio-Rad Laboratories, Richmond, CA).

### Proliferation assay

BxPC-3 cells (5 × 10^3^ per well) were seeded in 96-well plates and cultured overnight. Then, cells were treated with AR-42 at 0.2, 0.4, 0.6, 0.8, or 1 μM and incubated for 24 h. Proliferation of BxPC-3 cells was monitored by the incorporation of 5-bromo-2′-deoxyuridine (BrdU) using a cell proliferation ELISA kit (Roche, Mannhein, Germany) according to the manufacturer’s instructions. BrdU uptake was quantified using an ELISA reader at 590 nm (Bio-Rad).

### Cell cycle analysis

Cells (5 × 10^5^) were cultured for 12–18 h. For synchronizing cells at the G1/S phase, they were treated with 2 mM thymidine (Sigma-Aldrich) for 16 h. Afterwards, cells were washed by phosphate-buffered saline (PBS) to release them from thymidine block and grown in fresh medium with 10% FBS for 9 h. Subsequently, cells were subjected to another blocking experiment with the same concentration of thymidine for 10 h. After washing with PBS, cells were exposed to AR-42 at different concentrations and harvested after 24 h. Before staining with propidium iodide (PI, Sigma-Aldrich), cells were fixed overnight by 70% ethanol at 4°C. After centrifugation, the cell pellet was resuspended with PI (40 μg/mL), RNase A (50 μg/mL), and PBS in a total volume of 500 μL. Cells were incubated under shaking (50 rpm) at 37°C in the dark for 30 min. Immediately after the end of the incubation period, stained cells were analyzed by flow cytometry (FACScan, BD Immunocytometry Systems, San Jose, CA). DNA distribution was analyzed by Modfit (Verity Software House Inc., Topsham, ME) to determine the proportions of cells in different cell cycle phases.

### Western blot analysis

A total of 1 × 10^6^ cells was treated with AR-42 or DMSO (control) for 24 h. Cells were washed twice with ice-cold PBS, harvested, and disrupted in RIPA lysis buffer containing 1% protease inhibitor cocktail. Equal amounts of protein were separated using sodium dodecyl sulfate-polyacrylamide gel electrophoresis (SDS-PAGE) and transferred to nitrocellulose or polyvinylidene difluoride membranes. After blocking with 5% bovine serum albumin (BSA), primary antibodies against target proteins or β-actin were applied at 4°C overnight, followed by a horseradish peroxidase-conjugated secondary antibody for 1 h at room temperature. Protein bands were detected using ECL Western Blotting Detection System (GE Healthcare, Buckinghamshire, UK). Bands of β-actin served as loading control.

### Reactive oxygen species production assay

To evaluate the level of intracellular reactive oxygen species (ROS) production, BxPC-3 cells were stained by 2,7-dichlorodihydrofluorescein diacetate (DCFH-DA, Sigma-Aldrich) according to the manufacturer’s protocol. Cells (5 × 10^3^) in a P6 dish were pre-treated with 24 mM N-acetyl-L-cysteine (NAC) for 30 min [11] and then treated with either DMSO or AR-42 for 24 h. Then, the medium was removed, replaced by PBS containing 5% FBS and DCFH-DA, and incubated at 37°C in a humidified atmosphere of 95% air and 5% CO_2_ for 20 min. Treatment with 1.467 mM H_2_O_2_ for 10 min served as positive control. Cells were resuspended in PBS and analyzed by FACScan.

### Annexin V/PI assay

BxPC-3 cell death induced by AR-42 was determined by using an Alexa Fluor 488 Annexin V/Dead Cell Apoptosis Kit (Invitrogen, Carlsbad, CA) according to the manufacturer’s protocol. Drug-treated cells were stained with Annexin V and PI (5 μg/mL) and then analyzed by FACScan and ModFitLT v3.0 software (Becton Dickinson, Germany).

### Caspase 3 activation assay

Caspase-3 activity was determined using a fluorescein isothiocyanate (FITC) Active Caspase-3 Apoptosis Kit (BD Biosciences) according to the manufacturer’s instructions. Briefly, after treatment with AR-42 for 24 h, BxPC-3 cells were collected and incubated with Cytofix/Cytoperm solution at 4°C for 20 min. Thereafter, the solution was removed by centrifugation at 3,000 rpm for 5 min. Cells were then incubated with a FITC-conjugated monoclonal rabbit anti-active human-caspase-3 antibody for 30 min at room temperature. Cells were washed twice with PBS, and 500 μL of Perm/Wash buffer was added prior to flow cytometry.

### Confocal immunofluorescence microscopy

BxPC-3 cells were seeded onto microscopic cover glasses in a 24-well plate overnight and then treated with 1 μM AR-42 for 24 h. Cells were washed twice with PBS and fixed with 4% paraformaldehyde/10% Triton X-100 for 10 min at room temperature. Thereafter, cells were rinsed three times with PBS, blocked by 1% BSA, and subjected to immunofluorescence staining with an anti-AIF (1:100) antibody at 4°C overnight. Then, cells were washed with cold PBS and incubated with a donkey anti-mouse IgG-CFL 488 (1:100) secondary antibody at room temperature for 1 h. Finally, cells were mounted by using ibidi Mounting Medium with 4′,6-diamidino-2-phenylindole (Ibidi GmbH) and analyzed with a Confocal Microscope IX-81 (Olympus).

### Cell invasion assay

Matrigel (BD Bio, Germany) was added into Transwell inserts at a concentration of 1 mg/mL and incubated overnight at 37°C for consolidation. Cells (1 × 10^4^) mixed with serum-free RPMI-1640 medium containing AR-42 or DMSO were plated in the upper chamber, and 500 μL RPMI-1640 medium containing 10% FBS was added to the bottom chamber. After incubation at 37°C for 24 h, non-invasive cells were removed from the upper chamber with a cotton swab. Invasive cells that adhered to the inserted membranes were fixed with 4% formaldehyde for 30 min, permeabilized with 0.2% Triton X-100 for 10 min, and stained with Coomassie Blue. The number of invaded cells was counted in five random fields at 40× magnification using fluorescence microscopy. All experiments were repeated in triplicate.

### BxPC-3 xenograft tumor model

BALB/cAnN.Cg-*Foxnlnu*/CrlNarl male nude mice (6–8 weeks of age, BioLASCO Co., Ltd., Taiwan) were subcutaneously injected with 1 × 10^6^ BxPC-3 cells suspended in 50% Matrigel. As tumors became established (mean starting tumor volume, 135.9 ± 11.2 mm^3^), mice were randomly assigned to one of three groups (*n* = 6) that received the following treatment by oral gavage every other day: vehicle or AR-42 at 25 or 50 mg/kg of body weight. Mouse weights and tumor volumes (length × width^2^ × 0.52) were measured every other day. Mice were sacrificed when tumor volumes reached 1,000 mm^3^. The harvested tumors were weighted and photographed. All experimental procedures were performed in accordance with protocols approved by the Institutional Laboratory Animal Care and Use Committee of the Kaohsiung Medical University.

### Statistical analysis

All results are presented as the mean ± standard deviation (SD). Statistical analyses of control and treatment data were performed by using Student’s *t*-test. For *in vivo* study, differences among group means of tumor volumes and weight changes were analyzed using two-way ANOVAs followed by Tabular tests. All tests were 2-tailed and differences were considered to be significant if *P* < 0.05. Statistical analyses were performed using SPSS version 18 and Excel 2010.

## Results

### AR-42 inhibited pancreatic cancer cell proliferation by causing G2/M cell cycle arrest

**Fig 1:**
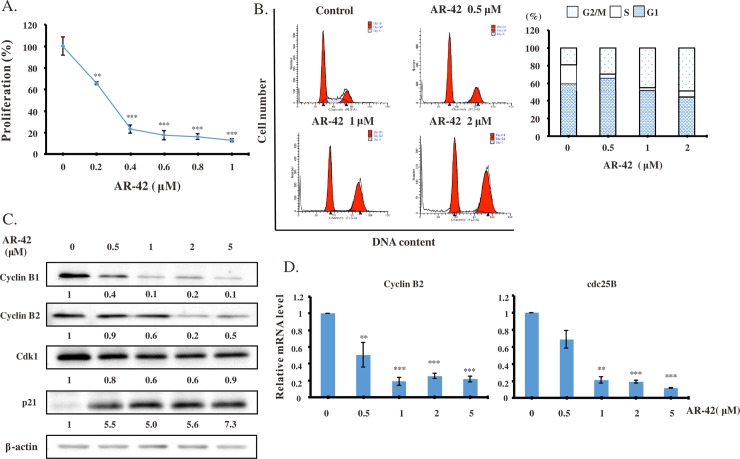
AR-42 inhibited pancreatic cancer cell growth and arrested cells at the G2/M phase. BxPC-3 or PANC-1 cells were exposed to different concentrations of AR-42 for 24 h. **(A)** BxPC-3 cell proliferation was analyzed by the 5-bromo-2′-deoxyuridine assay. **(B)** Distributions of PANC-1 cells in the G1, S, and G2/M stages of cell cycle after double thymidine block were determined by flow cytometry. **(C)** Expression levels of cell cycle-specific proteins in PANC-1 cells were determined by western blot analysis. The numbers under each blot represent values of corresponding band intensity relative to that of actin and untreated control. **(D)** Expression levels of cyclin B2 and cdc25B mRNA were examined by real-time PCR and normalized by mRNA levels of the reference gene *GAPDH*. Data are presented as the mean ± standard deviation of three independent experiments. Statistical significance of differences from mRNA levels in control experiments is indicated as follows: **P <0.*01*, ***P <0.*001*.

Cytotoxicity of AR-42 in six human pancreatic cancer cell lines (AsPC-1, SW1990, BxPC-3, COLO-357, MiaPaCa-2, and PANC-1) has been evaluated previously [22]. In our study, we used gemcitabine-responsive BxPC-3 and resistant PANC-1 cell lines as our testing model to examine the cell viability by the MTT assays (S1 Fig). Moreover, we showed that AR-42 concentration-dependently attenuated pancreatic cancer cell growth (Fig 1A). To unravel the effect of AR-42 on cell cycle, PANC-1 cells were synchronized at the G1/S phase by double thymidine block and incubated with 0, 0.5, 1, or 2 μM AR-42 for 24 h. We found that incubation with AR-42 concentration-dependently induced G2/M arrest (Fig 1B). The cell cycle is known to be regulated by cyclins and cyclin-dependent protein kinases (Cdks). Upon AR-42 treatment for 24 h, expression levels of the G2/M-specific proteins cyclin B1, cyclin B2, and Cdk1 were diminished, whereas Cdk inhibitor p21 expression was increased in PANC-1 cells (Fig 1C). Furthermore, AR-42 also decreased mRNA expression levels of cyclin B2 and cdc25B (Fig 1D). These data suggested that AR-42 could arrest pancreatic cancer cells at the G2/M phase.

### AR-42 induced ROS generation and DNA damage

**Fig 2:**
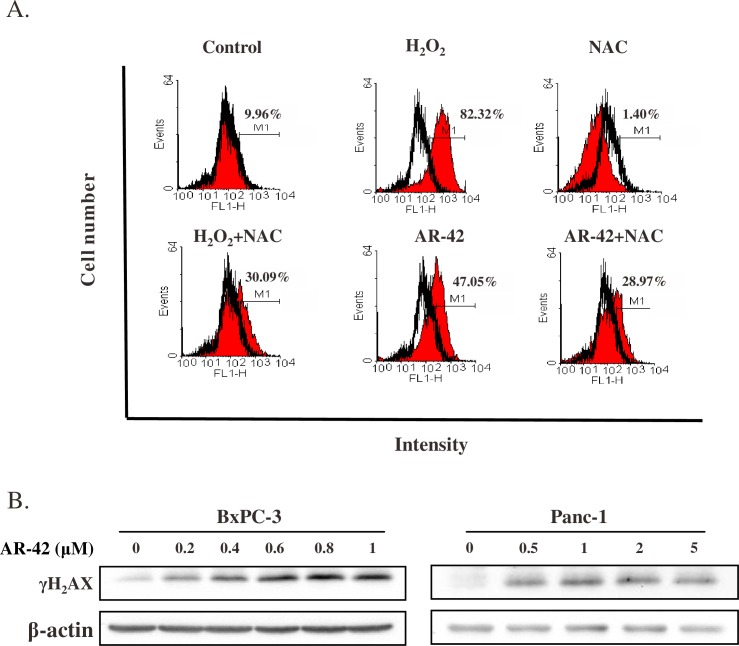
AR-42 induced ROS production and caused DNA damage in pancreatic cancer cells. **(A**) BxPC-3 cells were treated by 1 μM AR-42 for 24 h. Concentrations of H_2_O_2_ and NAC comprised 1.467 mM and 25 mM, respectively. ROS generation was analyzed by flow cytometry using 2,7-dichlorodihydrofluorescein diacetate. All data are representative of three independent experiments with similar results. **(B)** Expression of γH_2_AX was examined by western blot analysis.

Previous studies have implicated that ROS generation partially accounts for HDACi-mediated cell death [23,24]. As shown in Fig 2A, ROS generation was markedly increased by AR-42 in BxPC-3 cells, and such enhancement could partially be reversed by the ROS scavenger NAC. Increased ROS production causes DNA damage, particularly double strand breaks (DSBs) [25]. Upon treatment of PANC-1 and BxPC-3 cells with AR-42 for 24 h, protein expression levels of the DNA damage marker γH2AX were dramatically increased in both cell lines (Fig 2B), indicating that AR-42 promoted DSBs.

### AR-42 induced apoptosis in pancreatic cancer cells through caspase-dependent and caspase-independent pathways

**Fig 3:**
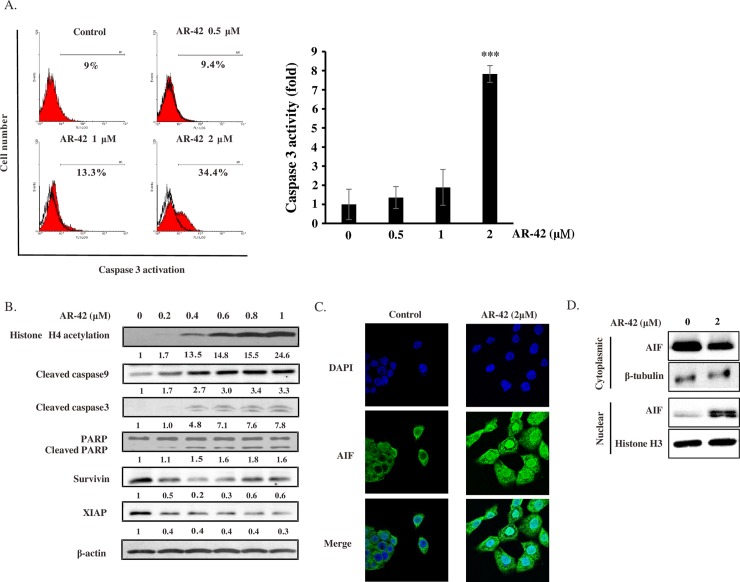
AR-42 induced apoptosis in pancreatic cancer cells via activating caspase 3-dependent and caspase 3-independent pathways. BxPC-3 cells were treated by indicated concentrations of AR-42 for 24 h. **(A)** Numbers of apoptotic BxPC-3 cells were determined by the caspase-3 activity assay. Fold increase in caspase-3 activation due to increasing doses of AR-42 treatment in comparison to control (control value = 1) are represented by the bar graph. Data represent mean ± SD from three independent experiments. *** P < 0.001 compared to the control group. **(B)** Expression levels of apoptosis-related proteins in BxPC-3 cells were determined by western blot analysis. The numbers under each blot represent values of corresponding band intensity relative to that of actin and untreated control. Translocation of apoptosis inducing factor (AIF) in BxPC-3 cells after treatment with 2 μM AR-42 for 24 h was detected by immunofluorescence **(C)** and western blot analysis of subcellular fraction **(D)**. β-tubulin and histone H3 served as markers of cytoplasmic and nuclear fractions respectively.

To investigate the involvement of apoptosis in AR-42-mediated cytotoxicity, expression levels of several apoptosis markers were examined. As shown in Fig 3A, AR-42 activated caspase-3 in a concentration-dependent manner. In addition, upregulation of apoptosis by AR-42 was evidenced by proteolytic cleavage of caspase-9, PARP, and caspase-3, as well as by a decrease in the levels of anti-apoptosis proteins survivin and XIAP. Notably, the levels of acetylated histone H4 were concentration-dependently increased by AR-42, confirming the inhibitory effect of the drug on HDAC (Fig 3B). In addition to inducing classical caspase-dependent apoptosis, AR-42 could also promote cell death by caspase-independent mechanisms. AIF is one of the important proteins that mediate caspase-independent cell death. AIF is normally located in the mitochondrial intermembrane space, and it causes large scale DNA fragmentation and caspase-independent apoptosis when it translocates from the mitochondria to the nucleus [26]. After AR-42 treatment, we found that in BxPC-3 cells, significant amounts of AIF were translocated from the mitochondria to the nucleus, indicating that AR-42 induced pancreatic cancer cell death both by caspase-dependent and caspase-independent apoptosis pathways (Fig 3C and 3D).

### Effects of AR-42 on expression levels of p53 mRNA, p53 protein, and p53-regulating miRNAs

**Fig 4:**
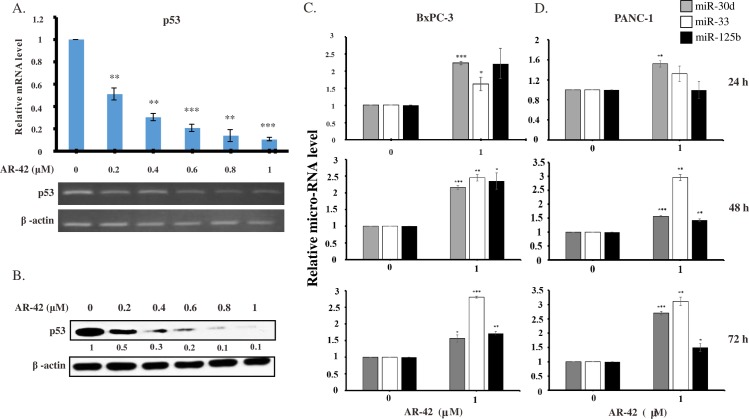
AR-42 decreased expression levels of p53 mRNA and protein and upregulated expression of p53 regulatory miRNAs in pancreatic cancer cells. BxPC-3 cells were treated with AR-42 for 24 h. **(A)** Expression levels of p53 mRNA were measured by real-time PCR (upper panel) and RT-PCR (lower panel). Data are presented as the mean ± standard deviation of three independent experiments. Statistical significance of differences from mRNA levels in control experiments is indicated as follows: **P <0.*01*, ***P <0.*001*. **(B)** Changes in p53 expression on the protein level were confirmed by western blot analysis. The numbers under each blot represent values of corresponding band intensity relative to that of actin and untreated control. Expression levels of miR-30d, miR-33, and miR-125b were determined by real-time PCR of AR-42 treated BxPC-3 **(C)** and PANC-1 **(D)** cells for 24, 48 and 72 h. Expression levels of miRNAs were normalized by that of the reference gene U6. Data are presented as the mean ± standard deviation of three independent experiments. Statistical significance of differences from miRNA levels in control experiments is indicated as follows: *P <0.*05*, **P <0.*01*, ***P <0.*001*.

To further investigate upstream regulation of AR-42-mediated apoptosis in BxPC-3 cells, we conducted a microarray assay. According to the microarray data (S1 and S2 Tables), we noticed that p53 expression was dramatically changed after AR-42 treatment. To evaluate the impact of AR-42 on p53 expression in more detail, BxPC-3 cells were treated with 0, 0.2, 0.4, 0.6, 0.8, 1 μM AR-42 for 24 h. Expression levels of p53 mRNA and protein were significantly decreased upon AR-42 treatment in a concentration-dependent manner (Fig 4A and 4B). A decrease in p53 mRNA expression by AR-42 could be mediated through the regulation of upstream modulators of p53. MicroRNA-30d (miR-30d), miR-33, and miR-125b have been shown to inhibit p53 mRNA expression [27]. In our study, we found that AR-42 upregulated miR-30d, miR-33, and miR-125b in both BxPC-3 (Fig 4C) and PANC-1 (Fig 4D) cells, which suggested that AR-42 suppresses p53 expression by inducing the expression of several p53-targeting miRNAs.

### AR-42 reduced pancreatic cancer cell aggressiveness

**Fig 5:**
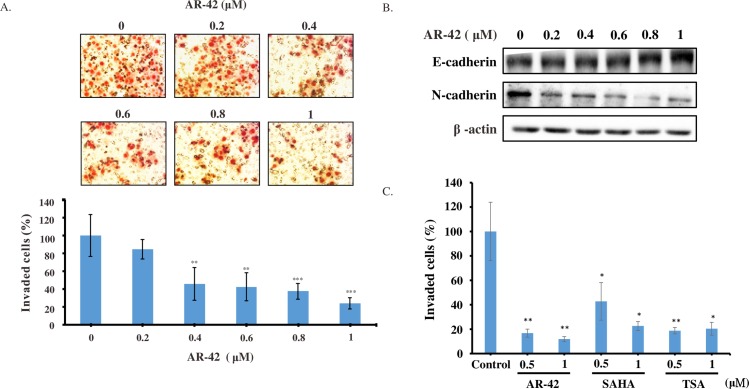
AR-42 suppressed BxPC-3 cell invasiveness. (A) Cells invading Matrigel after 24 h were detected as stained cells on the opposite side of membrane. Top panel illustrates representative photomicrographs of cells treated with indicated concentrations of AR-42. Bottom panel illustrates quantitative analysis of invasion ability depending on AR-42 concentration. The ability of BxPC-3 cells to invade Matrigel was significantly attenuated by AR-42 in a concentration-dependent manner compared to that of DMSO-treated control cells. (B) The expression of E- and N-cadherin were determined by western blot analysis after BxPC-3 cells were treated with AR-42 for 24 h. Results were confirmed by repeated experiments. (C) The comparison of the inhibitory effects of AR-42, SAHA and TSA on invasiveness of BxPC-3 cells. Data of (A, C) are presented as the mean ± standard deviation of three independent experiments. Statistical significance of differences from the percentage of invasive cells in control experiments is indicated as follows: *P <0.*05*, **P <0.*01*, ***P <0.*001*.

Given that the invasion of cancer cells is an essential step for tumor metastasis, we further investigated whether AR-42 had any impact on cell invasiveness. As shown in Fig 5A, the invasiveness of BxPC-3 cells was dramatically suppressed by AR-42. Epithelial cells use E-cadherin as a major protein in adherens junctions and promote its interaction with the extracellular domain of another E-cadherin molecule from a neighboring cell. During Epithelial-to-Mesenchymal Transition, the activity of the adherens junctions is substantially modified, predominantly owing to the replacement of E-cadherin by N-cadherin, a process called “cadherin switching”. As shown in Fig 5B, AR-42 increased E-cadherin and downregulated N-cadherin expression level. These results suggest that AR-42 suppresses the invasion of BxPC-3 pancreatic cancer cells through tuning the cadherin switching. In addition, AR-42 showed greater potency in suppressing the invasiveness of BxPC-3 cells than other pan-HDAC inhibitors, suberoylanilide hydroxamic acid (SAHA) and trichostatin A (TSA) (Fig 5C). These data implied that AR-42 has an anti-metastasis potential and can be used for treating pancreatic cancer.

### AR-42 significantly diminished BxPC-3 xenograft tumor growth *in vivo*

**Fig 6:**
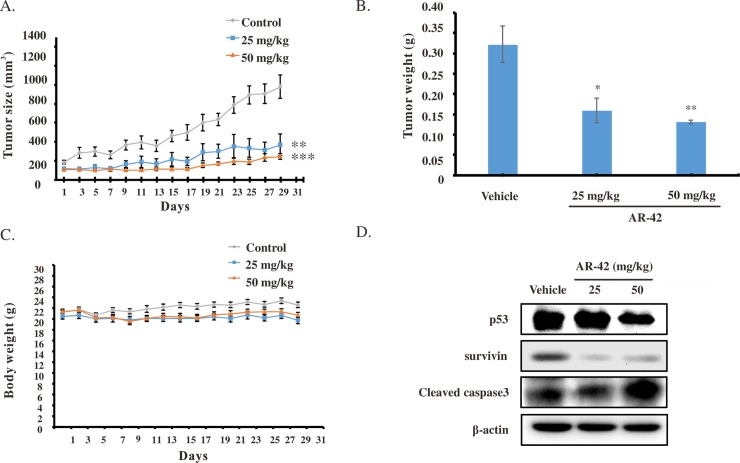
*In vivo* efficacy of AR-42 in BxPC-3 xenografted mice. **(A)** BxPC-3 xenograft tumor volumes in mice treated via oral gavage with either vehicle or AR-42 (25, 50 mg/kg) every other day for 29 days. **(B)** Representative tumors from animals treated with vehicle or AR-42 (25, 50 mg/kg) **(C)** Body weight of xenografted mice in vehicle- and AR-42-treated groups. Data are presented as the mean ± standard deviation of measurements in 6 mice per group. Statistical significance of differences from the values in control group is indicated as follows: *P <0.*05*, **P <0.*01*, ***P <0.*001*. (D) Western blot showing the expression of p53, survivin and cleaved caspase-3 proteins as analyzed from tumor protein extracts in each treatment group.

To further clarify anticancer activity of AR-42 with regard to its potential clinical use, we examined its antitumor effects *in vivo*. Male BALB/c NU mice (6–8 weeks old) were given subcutaneous injections of pancreatic cancer BxPC-3 cells, followed by oral administrations of vehicle or AR-42 at 25 and 50 mg/kg body weight every other day for 29 days. As shown in Fig 6, AR-42 significantly suppressed tumor growth in BxPC-3 tumor-bearing mice (Fig 6A) and AR-42-treating tumors weighed significantly less (reduction by 40–60%) than vehicle control tumors (Fig 6B). No significant impacts on mouse body weights were observed at the end point of the study (Fig 6C), which indicated that no overt toxicity signs were noted in any of the treated mice. These data suggested that AR-42 potently suppressed pancreatic cancer growth *in vivo*. Treatment with AR-42 resulted in the decrease of p53, survivin and increase of cleaved caspase-3 (Fig 6D), confirming the *in vitro* results.

## Discussion

**Fig 7:**
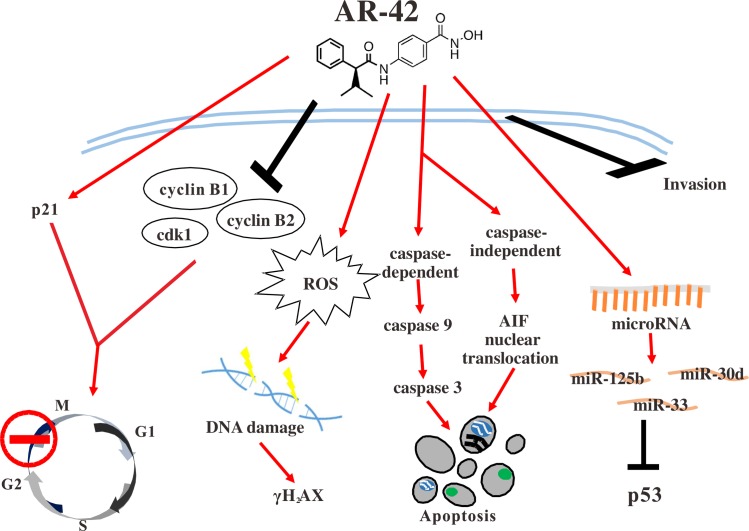
Schematic representation of multiple mechanisms affected by AR-42 during inhibition of pancreatic cancer cell growth.

HDACis have been reported to exert anticancer activity by regulating various important biochemical pathways, such as apoptosis and cell cycle [28–30]. In addition, HDACis altered expression of genes such as p21 through promoter chromatin acetylation [31]. Although anti-tumor effects of AR-42 have been demonstrated in many cancers, including prostate cancer, ovarian cancer, multiple myeloma, schwannoma, and meningioma [17–19,32], AR-42 activity in pancreatic cancer has not been elucidated in detail. In this study, we demonstrated that AR-42 inhibited growth of pancreatic cancer cells *in vitro* and *in vivo*, and caused cell cycle G2/M arrest by regulating expression levels of cyclin B1, cyclin B2, CDK1, and p21 (Fig 1). AR-42 also induced ROS generation, which caused DNA damage, and activated both caspase-dependent and caspase-independent pathways, triggering apoptosis of pancreatic cancer cells (Figs 2 and 3). In addition, AR-42 regulated expression levels of the important tumor suppressor gene p53, probably via affecting its upstream regulation by several miRNAs (Fig 4C). Furthermore, AR-42 inhibited cell invasiveness *in vitro* and suppressed tumor growth *in vivo* (Figs 5 and 6). We therefore suggest that anti-tumor activity of AR-42 in pancreatic cancer is mediated by multiple pathways (Fig 7).

Recent studies implicated ROS production as a promising strategy in anticancer therapy [33–35]. Here, we showed that AR-42 elevated ROS levels in pancreatic cancer cells, which was similar to the effects of other HDACis in different cell types [36,37]. ROS production induced by AR-42 was only partially reversed by the ROS scavenger NAC (Fig 2A), indicating that AR-42 also induced other sources of ROS (*e*.*g*., iNOS, or xanthine oxidase), which caused DNA damage or cell death [38,39]. However, AR-42 did not induce ROS in acute myelogenous leukemia [5], suggesting that upregulation of ROS production by AR-42 might be a tumor type-specific phenomenon. Accumulating evidence indicates that HDACis affect DNA repair systems. For example, suberoylanilide hydroxamic acid has been demonstrated to downregulate the expression of DNA repair Rad51 protein in cancers such as osteosarcoma and rhabdomyosarcoma [40,41]. Another study revealed that the adamantyl-hydroxamate HDACi H6CAHA decreased *ATM* gene expression and impaired ATM protein phosporylation [41]. Our data showed that AR-42 significantly upregulated DNA damage marker γH_2_AX protein expression (Fig 2B), confirming that AR-42 caused DNA damage in pancreatic cancer cells as well. NAC is a well-known antioxidant agent, which was reported to inhibit ROS-dependent apoptosis [42,43]. NAC anti-ROS activity is mediated via upregulation of glutathione levels in cells or by an increase in the redox potential of thiols [44]. Unexpectedly, a combined treatment of AR-42 and NAC did not reverse but further enhanced cell death and DNA damage in pancreatic cancer cells (S2 Fig). According to a previous study, however, NAC induced hypoxic apoptosis in mouse embryonic fibroblasts, MIA PaCa-2 human pancreatic cancer cells, and A549 human lung carcinoma cells [45]. Because NAC is a glutathione precursor, enhanced cell death caused by this substance might be due to increased glutathione level [34]. Dysregulated redox status of cells may lead to the inhibition of important biochemical cascades, such as NFκB signaling pathway, which could explain why a combination of AR-42 with NAC increased cell toxicity. According to our findings, taking antioxidant dietary supplements simultaneously with AR-42 may be a more potent therapeutic strategy in fighting pancreatic cancer.

AIF is type-I inner mitochondrial membrane protein with the N-terminus facing the matrix and the C-terminal portion residing in the mitochondrial intermembrane space [46,47]. During apoptosis, AIF is cleaved by a proteolytic processing and released from mitochondria, the nuclear localization signal motif in the protein allows it to translocate to the nucleus where it interacts with DNA and leads to chromatin condensation and DNA degradation into 50 kb fragments. AIF release-inducing cell death is caspase-independent, which often was accompanied by a lower mitochondrial membrane potential [48]. Opening of the mitochondrial permeability transition pore has been demonstrated to induce depolarization of the transmembrane potential. In some apoptotic systems, the loss of transmembrane potential is a consequence of the apoptotic-signaling pathway but not an early requirement [49]. By JC-1 staining, we observed the depolarization of the transmembrane potential of BxPC-3 cells with prolonged treatment of AR-42 (S3 Fig). AIF-dependent killing pathway plays a major role in the death of certain cancer types such as NSCLC [50]. However, this caspase-independent apoptosis is not a common pathway been mentioned in pancreatic cancer. Our study demonstrated that AR-42 could induce AIF nuclear translocation and subsequently caused pancreatic cancer cells death.

P53 is a well-known tumor suppressor that senses different stresses encountered by cells and protects cells from injury by initiating various biochemical cascades [51]. Dysfunction of p53-mediated protection by mutation or depletion of p53 is common in many cancers [52]. The majority of pancreatic cancer cells bear p53 point mutations, including the cell lines used in this study [53]. Our data demonstrated that expression levels of p53 mRNA and protein were dramatically decreased upon AR-42 treatment, suggesting that AR-42 could decrease deleterious effects of mutant p53 in pancreatic cancer cells (Fig 4A and 4B), and such inhibitory effect was greater than other pan-HDAC inhibitors, TSA and SAHA (S4 Fig). Previous studies indicated that many anticancer therapeutic agents exhibit antitumor potency by decreasing mutant p53 activity or restoring functions of normal p53 [52]. Characterization of the relationship between AR-42 and mutant p53 is crucial for elucidating how AR-42 exerts its anticancer effects via p53 regulation in pancreatic cancer. Interestingly, Stojanovic *et al*. just reported that HDAC1 and HDAC2 could integrate the expression of p53 mutants in pancreatic cancer [54]. Their data revealed that the class I HDAC inhibitors, as well as the specific genetic elimination of HDAC1 and HDAC2, reduce the expression of mutant p53 mRNA and protein levels. They found that HDAC1, HDAC2 and MYC directly bind to the TP53 gene and that HDAC1/HDAC2-mediated MYC recruitment drops upon HDAC inhibitor treatment. Their results illustrated a previously unrecognized class I HDAC-dependent control of the p53 gene and provided evidence for a contribution of MYC. AR-42 is a broad-spectrum class I and II HDAC inhibitor; it is plausible that AR-42 decreased the mutant p53 expression in the similar way as Stojanovic *et al*. reported. Meanwhile, according to our results, AR-42 upregulated expression levels of three miRNA, miR-30d, miR-33, and miR-125b (Fig 4C and 4D), which have been previously shown to inhibit p53 gene expression. MiR-33 inhibited expression of Cdk6 and cyclin D1 in liver cells [55], and we demonstrated that AR-42 decreased mRNA expression of cyclin B2 and cdc25B, suggesting that miR-33 might be involved in AR-42-mediated cell cycle regulation. An increasing number of studies show that pancreatic cancer requires autophagy for tumor growth, and higher autophagic activity was detected in pancreatic cancer cells [56,57]. Furthermore, miR-30d has been shown to repress the expression of important autophagy genes *BECN1*, *BNIP3L*, *ATG12*, *ATG5*, and *ATG2*. MiR-30d also inhibited autophagosome formation and conversion of LC3B-I to LC3B-II in different cancers [58]. Upregulation of miR-30d by AR-42 suggested an important clue about the interplay between autophagy and p53 expression in pancreatic cancer cell progression. Further experiments to decipher the mechanisms underlying the relationships between AR-42, miRNAs, p53, and autophagy are currently underway in our laboratory.

Recently, Henderson *et al*. showed that AR-42 suppressed tumor growth and muscle wasting in the KP^fl/fl^C transgenic mice model of pancreatic cancer [21]. Moreover, a combination of AR-42 and gemcitabine led to a significantly longer survival of transgenic mice, compared to the viability of animals treated by either agent alone. In line with our *in vitro* data, we found that a combination of AR-42 and gemcitabine had a significantly greater and synergistic cytotoxic effect on pancreatic cancer cells than the effects of the two drugs used separately (S5 Fig). Alternative strategies targeting other pathways and taking advantage of drug combination effects might be a new perspective in clinical practice than can help to overcome the obstacles in pancreatic cancer therapy caused by gradually increasing resistance to chemotherapeutic drugs and gemcitabine side effects.

In summary, we have demonstrated that AR-42 exhibited strong cytotoxic activity against pancreatic cancer cells at low submicromolar concentrations, which was mediated by multiple biochemical mechanisms. Therefore, AR-42 may be a therapeutically promising drug for pancreatic cancer treatment.

## Supporting information

S1 Text Supporting Materials and Methods; Supporting References. (DOCX)

S1 Table Genes overexpressed with ≧1.5 fold-change in AR-42-treated BxPC-3 cells. (DOCX)

S2 Table Genes down-regulated with ≧1.5 fold-change in AR-42-treated BxPC-3 cells. (DOCX)

S1 Fig Effects of AR-42 on cell viability of 2 pancreatic cancer cell lines. (PPTX)

S2 Fig The AR-42-mediated cytotoxicity and DNA damage could not be restored by NAC. (PPTX)

S3 Fig Effects of AR-42 treatment on mitochondial membrane potential (MMP) in BxPC-3 cells. (PPTX)

S4 Fig AR-42, SAHA and TSA decreased expression levels of p53 mRNA and protein in pancreatic cancer cells. (PPTX)

S5 Fig Isobolograms showing the combination of AR-42 and gemcitabine for both BxPC-3 cells. (PPTX)
