# Supplementary material for: Novel histone deacetylase inhibitor AR-42 exhibits antitumor activity in pancreatic cancer cells by affecting multiple biochemical pathways
Source: PLoS One. 2017 Aug 22;12(8):e0183368. doi: 10.1371/journal.pone.0183368 (PMC5567660; doi:10.1371/journal.pone.0183368)
Supplement: S1 Text — (DOCX) [file pone.0183368.s001.docx]

**SupPORTING TEXT**

**Novel histone deacetylase inhibitor AR-42 exhibits antitumor activity in pancreatic cancer cells by affecting multiple biochemical pathways**

Yi-Jin Chen, Wen-Hung Wang, Wan-Yu Wu, Chia-Chi Hsu, Ling-Rung Wei, Sheng-Fan Wang, Ya-Wen Hsu, Chih-Chuang Liaw, and Wan-Chi Tsai^*^

**SUPPORTING MATERIALS AND METHODS**

Microarray analysis

BxPC-3 cells were treated with 1 μM AR-42 or DMSO (control) for 24 h. Total RNA was isolated by Trizol reagent (Invitrogen) according to the manufacturer’s protocol. RNA (1 μg) was shipped frozen to Phalanx Biotech Group, Taiwan for labeling and hybridization. Eberwine-based amplification method with Amino Allyl MessageAmp II aRNA Amplification Kit (Ambion, AM1753) were used to generate amino-allyl antisense RNA(aa-aRNA). Labeled aRNA coupled with NHS-CyDye was prepared and purified prior to hybridize to Human OneArray® (Array Version HOA 6.1). Data were analyzed with Rosetta Resolver® System (Rosetta Biosoftware). Standard selection criteria to identify differentially expressed genes are established at log2 |Fold change| ≥ 1.5 and P < 0.05.

RNA isolation and RT-PCR

Total RNA and miRNA were isolated by Trizol reagent (Invitrogen, Carlsbad, CA) according to the manufacturer’s protocol. One µg of total RNA was subjected to reverse transcription for cDNA synthesis. The reverse transcription of miRNA was performed by miScript PCR Starter Kit (Qiagen, Hilden, Germany). The cDNA was diluted by DEPC-water for appropriate concentration. The cDNA reaction mixtures contain gene-specific primer. The PCR condition was 94 °C (30 s), 64 °C (30 s), and 72 °C (30 s) for 33 cycles. The primers sequences of actin and p53 are as follows:

| Target gene | Sequences |
| --- | --- |
| actin | F: 5’-GAGTCAACGGATTTGGTCGT-3’  R: 5’-TTGATTTTGGAGGGATCTCG-3’ |
| p53 | F: 5’-GTTCCGAGAGCTGAATGAGG-3’  R: 5’-TCTGAGTCAGGCCCTTCTGT-3’ |

Real-Time PCR

A final 15 µl reaction mixture contained cDNA, FastStart Universal SYBR Green Master (Roche) and gene-specific primers. The primers sequences used in real-time PCR were as follows:

| Target gene | Sequences |
| --- | --- |
| Cyclin B2 | F: 5’-TCATCCTTCTAAGGTAGCAG-3’  R: 5’-TACTGCTGCTTTAAGTTCCA-3’ |
| survivin | F: 5’-ACCGCATCTCTACATTCAAG-3’  R: 5’-CAAGTCTGGCTCGTTCTC-3’ |
| XIAP | F: 5’-CCGTGCGGTGCTTTAGTTGT-3’  R: 5’-TTCCTCGGGTATATGGTGTC-3’ |
| Cdc25B | F: 5’-TCATTTTCCACTGTGAATTCTCATC-3’  R: 5’-AGTCGTTGACAGCACGGTCTC-3’ |
| GAPDH | F: 5’-GAGTCAACGGATTTGGTCGT-3’  R: 5’-TTGATTTTGGAGGGATCTCG-3’ |

The quantitation of target genes was performed using ABI 7500 Real-Time PCR System (Applied Biosystems, CA).

Reverse transcription and Quantification of miRNA

MicroRNA was analyzed with miScript PCR Starter Kit (Qiagen) and Primer assays (Qiagen) according to the manufacturer’s protocol. The quantitation of target miRNA was by ABI 7500 Real-Time PCR System.

Subcellular fractionation

Twenty-four hours DMSO or 2 μM AR-42-treated BxPC-3 cells were harvested. Cells were trypsinized and centrifuged at 500 g for 5 min at 4 °C. The isolation of cytoplasmic and nuclear proteins was followed by the manufacturer’s instruction of Nuclear & Cytoplasmic Extraction kit (G-Biosciences, St Louis, MO).

MTT assays

For cell growth analysis cells were seeded in 96-well flat-bottom plates (5 × 10^3^/well) and treated with various concentrations of test agents for 24 h. To quantify cell viability, old medium was replaced with 150 μL fresh medium containing 10% MTT solution (Sigma-Aldrich). After 1 h incubation at 37 °C, the MTT solution was removed and the intracellular formazan crystals were solubilized with 100 μL DMSO. The absorbance levels for each sample at 595 nm were measured using the microplate reader (Bio-Rad Laboratories, Richmond, CA). The data were obtained from six replicates.

Immunoblotting

BxPC-3 cells (1.5 × 10^5^/mL) were treated with DMSO or AR-42 with or without NAC for 24 h and washed twice with PBS. The total cell lysates were collected and applied for the further detection.

Mitochondrial membrane potential

Mitochondrial membrane potential was quantified by flow cytometry using a MitoProbe JC-1 Assay Kit (Life Technologies). Cells were treated with DMSO (control), AR-42 (0.5, 1, or 2 μM), or 25 μM clioquinol with 10 mM copper as a positive control. After 24 h, cells were collected and incubated with 200 μM JC-1 dye for 30 minutes at 37 °C. Cells were centrifuged at 1300 rpm for 5 minutes to remove supernatant and resuspended in 1 mL PBS prior to flow cytometry.

Analysis of combined drug effect

Drug synergy was determined with isobolograms, based on the median-effect principle described by Chou and Talalay [1]. Effects of individual or combined treatment with AR-42 and/or gemcitabine on BxPC-3 cells for 48 h were determined by MTT assays and analyzed with isobolograms using CalcuSyn software (v 1.0) (Biosoft, Ferguson, MO). CalcuSyn analyzed the dose effect of AR-42 (0.125, 0.25, 0.5, 1 μM) and gemcitabine (0.025, 0.05, 0.1, 0.2 μM) in BxPC-3. Dose effects of AR-42 and gemcitabine with fixed dose ratios were calculated using a combination index (CI) that identified dual-drug synergism, antagonism, or additivity. The CI values were determined to three categories: CI <1🡪 synergism (the dose effect point below the diagonal line), CI =1🡪 additive (the dose effect point on the diagonal line); and CI >1🡪 antagonism (the dose effect point over the diagonal line).

**SUPPORTING REFERENCES**

1. Chou TC, Talalay P. Quantitative analysis of dose-effect relationships: the combined effects of multiple drugs or enzyme inhibitors. Advances in Enzyme Regulation. 1984;22:27-55.
