# Supplementary material for: Novel histone deacetylase inhibitor AR-42 exhibits antitumor activity in pancreatic cancer cells by affecting multiple biochemical pathways
Source: PLoS One. 2017 Aug 22;12(8):e0183368. doi: 10.1371/journal.pone.0183368 (PMC5567660; doi:10.1371/journal.pone.0183368)
Supplement: S1 Table — (DOCX) [file pone.0183368.s002.docx]

**S1 Table. Genes overexpressed with ≧1.5 fold-change in AR-42-treated BxPC-3 cells**

| **Gene Symbol** | **Description** | **Fold change** | **p-value** |
| --- | --- | --- | --- |
| **GPR84** | **G protein-coupled receptor 84** | **2.644183** | **3.99E-18** |
| **CYP1A1** | **cytochrome P450, family 1, subfamily A, polypeptide 1** | **2.408629** | **1.02E-19** |
| **GPR18** | **G protein-coupled receptor 18** | **2.302412** | **5.29E-20** |
| **ANKRD22** | **ankyrin repeat domain 22** | **2.235822** | **1.16E-18** |
| **CD38** | **CD38 molecule** | **1.99277** | **2.24E-16** |
| **CCL2** | **chemokine (C-C motif) ligand 2** | **1.883721** | **7.61E-13** |
| **IER3** | **immediate early response 3** | **1.860707** | **0.000045** |
| **CCL2** | **chemokine (C-C motif) ligand 2** | **1.814568** | **2.06E-17** |
| **ZNF264** | **zinc finger protein 264** | **1.745589** | **0.000026** |
| **ALOX5AP** | **arachidonate 5-lipoxygenase-activating protein** | **1.740822** | **1.99E-20** |
| **CCL20** | **chemokine (C-C motif) ligand 20** | **1.599742** | **6.64E-10** |
| **PRL** | **prolactin** | **1.572641** | **1.64E-13** |
| **S100B** | **S100 calcium binding protein B** | **1.532995** | **0.030956** |
| **RETN** | **resistin** | **1.508522** | **4.04E-11** |
| **CLEC5A** | **C-type lectin domain family 5, member A** | **1.504468** | **0.000001** |
