# Supplementary material for: Novel histone deacetylase inhibitor AR-42 exhibits antitumor activity in pancreatic cancer cells by affecting multiple biochemical pathways
Source: PLoS One. 2017 Aug 22;12(8):e0183368. doi: 10.1371/journal.pone.0183368 (PMC5567660; doi:10.1371/journal.pone.0183368)
Supplement: S2 Table — (DOCX) [file pone.0183368.s003.docx]

**S2 Table. Genes down-regulated with ≧1.5 fold-change in AR-42-treated BxPC-3 cells**

| **Down-regulated genes** | | | |
| --- | --- | --- | --- |
| **Gene Symbol** | **Description** | **Fold change** | **p-value** |
| **CD70** | **CD70 molecule** | **-2.51736** | **1.52E-20** |
| **SREBF1** | **sterol regulatory element binding transcription factor 1** | **-2.355079** | **1.59E-14** |
| **CD79B** | **CD79b molecule, immunoglobulin-associated beta** | **-2.28707** | **8.12E-14** |
| **LASS1** | **LAG1 homolog, ceramide synthase 1** | **-2.152388** | **4.23E-16** |
| **MMP9** | **matrix metallopeptidase 9 (gelatinase B, 92kDa gelatinase, 92kDa type IV collagenase)** | **-2.101386** | **1.34E-10** |
| **KANK4** | **KN motif and ankyrin repeat domains 4** | **-2.072707** | **1.08E-09** |
| **PRDX2** | **peroxiredoxin 2** | **-2.066833** | **6.60E-12** |
| **OLIG1** | **oligodendrocyte transcription factor 1** | **-2.060971** | **1.98E-11** |
| **HS3ST2** | **heparan sulfate (glucosamine) 3-O-sulfotransferase 2** | **-2.04444** | **1.36E-09** |
| **SCD** | **stearoyl-CoA desaturase (delta-9-desaturase)** | **-1.842059** | **0.000002** |
| **SNTB2** | **syntrophin, beta 2 (dystrophin-associated protein A1, 59kDa, basic component 2)** | **-1.785251** | **1.63E-08** |
| **CAB39L** | **calcium binding protein 39-like** | **-1.732699** | **9.12E-13** |
| **PRDX2**  **GHRL** | **peroxiredoxin 2**  **ghrelin/obestatin prepropeptide** | **-1.700752**  **-1.627456** | **0.000015**  **1.58E-12** |
| **PRDX2** | **peroxiredoxin 2** | **-1.595204** | **3.45E-12** |
| **TP53INP2** | **tumor protein p53 inducible nuclear protein 2** | **-1.588634** | **2.36E-10** |
| **LMO1** | **LIM domain only 1 (rhombotin 1)** | **-1.570884** | **0.000001** |
| **PRKCB** | **protein kinase C, beta** | **-1.554443** | **0.000001** |
| **C14orf1** | **chromosome 14 open reading frame 1** | **-1.533462** | **1.15E-09** |
