# Supplementary material for: Novel histone deacetylase inhibitor AR-42 exhibits antitumor activity in pancreatic cancer cells by affecting multiple biochemical pathways
Source: PLoS One. 2017 Aug 22;12(8):e0183368. doi: 10.1371/journal.pone.0183368 (PMC5567660; doi:10.1371/journal.pone.0183368)
Supplement: S1 Fig — (PPTX) [file pone.0183368.s004.pptx]

## Slide 1
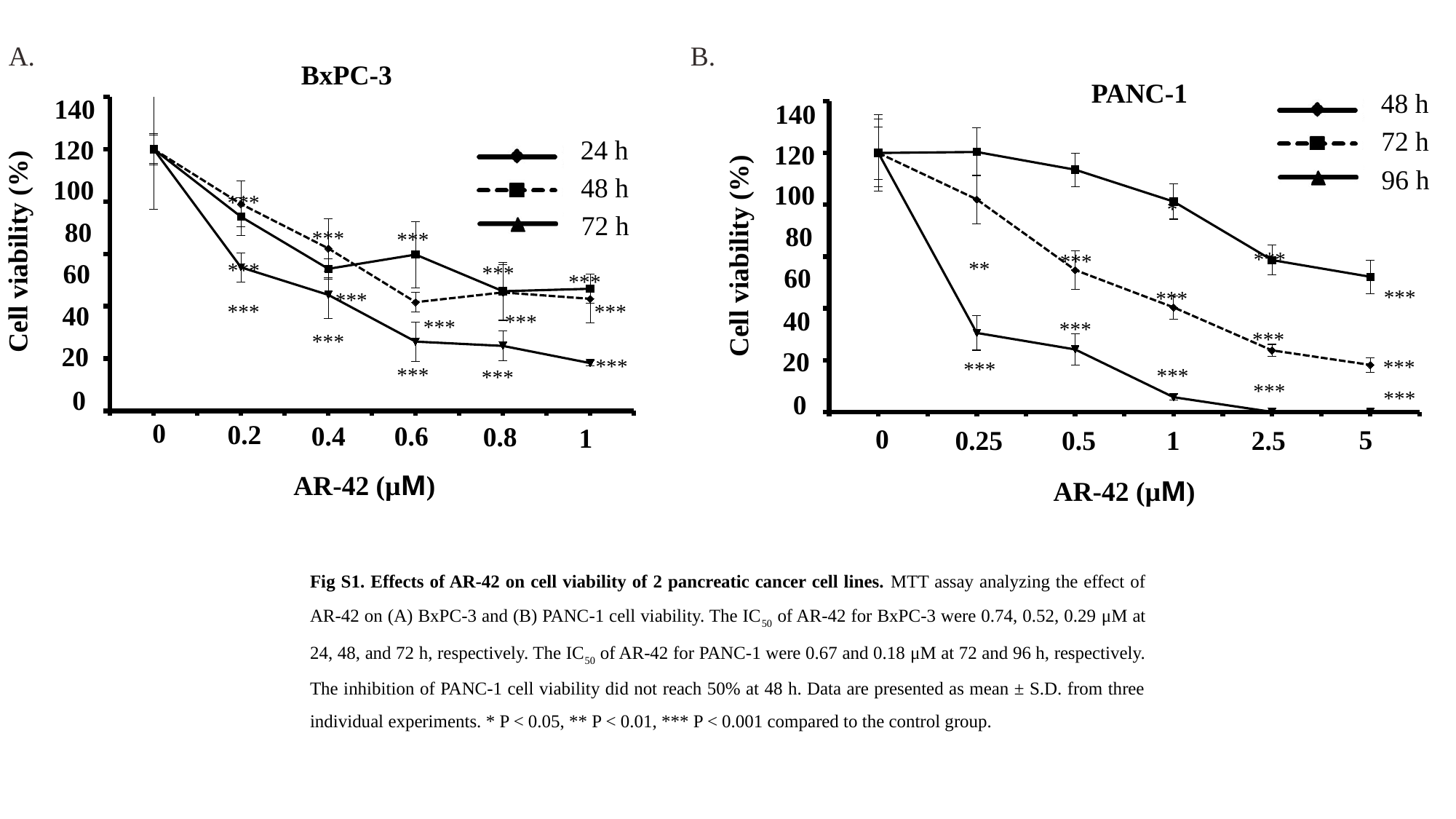

A.
B.
BxPC-3
PANC-1
48 h
140
### Chart
| Category | 24h | 48h | 72h |
|---|---|---|---|
| 0 | 1.0 | 1.0 | 1.0 |
| 200 | 0.7418351477449454 | 0.7903225806451614 | 0.5484931506849313 |
| 400 | 0.5427682737169517 | 0.620967741935484 | 0.44328767123287666 |
| 600 | 0.5972006220839813 | 0.4153225806451613 | 0.26465753424657534 |
| 800 | 0.4572317262830482 | 0.45262096774193555 | 0.2482191780821918 |
| 1000 | 0.4665629860031104 | 0.4284274193548387 | 0.1824657534246575 |140
### Chart
| Category | 48h | 72h-IC50: 0.833 μM | 96h-IC50: 0.0714 μM |
|---|---|---|---|
| 0 | 1.0 | 1.0 | 1.0 |
| 0.25 | 1.0038659793814433 | 0.820962888665998 | 0.3049645390070922 |
| 0.5 | 0.9355670103092782 | 0.5481444332998997 | 0.24113475177304966 |
| 1 | 0.8125 | 0.40371113340020065 | 0.056737588652482275 |
| 2.5 | 0.5863402061855669 | 0.23771313941825478 | 0.0 |
| 5 | 0.5219072164948453 | 0.18154463390170517 | 0.0 |72 h
24 h
120
120
96 h
48 h
100
100
***
*
72 h
80
Cell viability (%)
80
Cell viability (%)
***
***
***
***
**
60
***
***
60
***
***
***
***
***
***
40
40
***
***
***
***
***
20
20
***
***
***
***
***
***
***
0
***
0
0
0.2
0.4
0.6
0.8
1
0
5
0.5
1
2.5
0.25
AR-42 (μM)
AR-42 (μM)
Fig S1. Effects of AR-42 on cell viability of 2 pancreatic cancer cell lines. MTT assay analyzing the effect of AR-42 on (A) BxPC-3 and (B) PANC-1 cell viability. The IC50 of AR-42 for BxPC-3 were 0.74, 0.52, 0.29 μM at 24, 48, and 72 h, respectively. The IC50 of AR-42 for PANC-1 were 0.67 and 0.18 μM at 72 and 96 h, respectively. The inhibition of PANC-1 cell viability did not reach 50% at 48 h. Data are presented as mean ± S.D. from three individual experiments. * P < 0.05, ** P < 0.01, *** P < 0.001 compared to the control group.
