# Supplementary material for: Novel histone deacetylase inhibitor AR-42 exhibits antitumor activity in pancreatic cancer cells by affecting multiple biochemical pathways
Source: PLoS One. 2017 Aug 22;12(8):e0183368. doi: 10.1371/journal.pone.0183368 (PMC5567660; doi:10.1371/journal.pone.0183368)
Supplement: S2 Fig — (PPTX) [file pone.0183368.s005.pptx]

## Slide 1
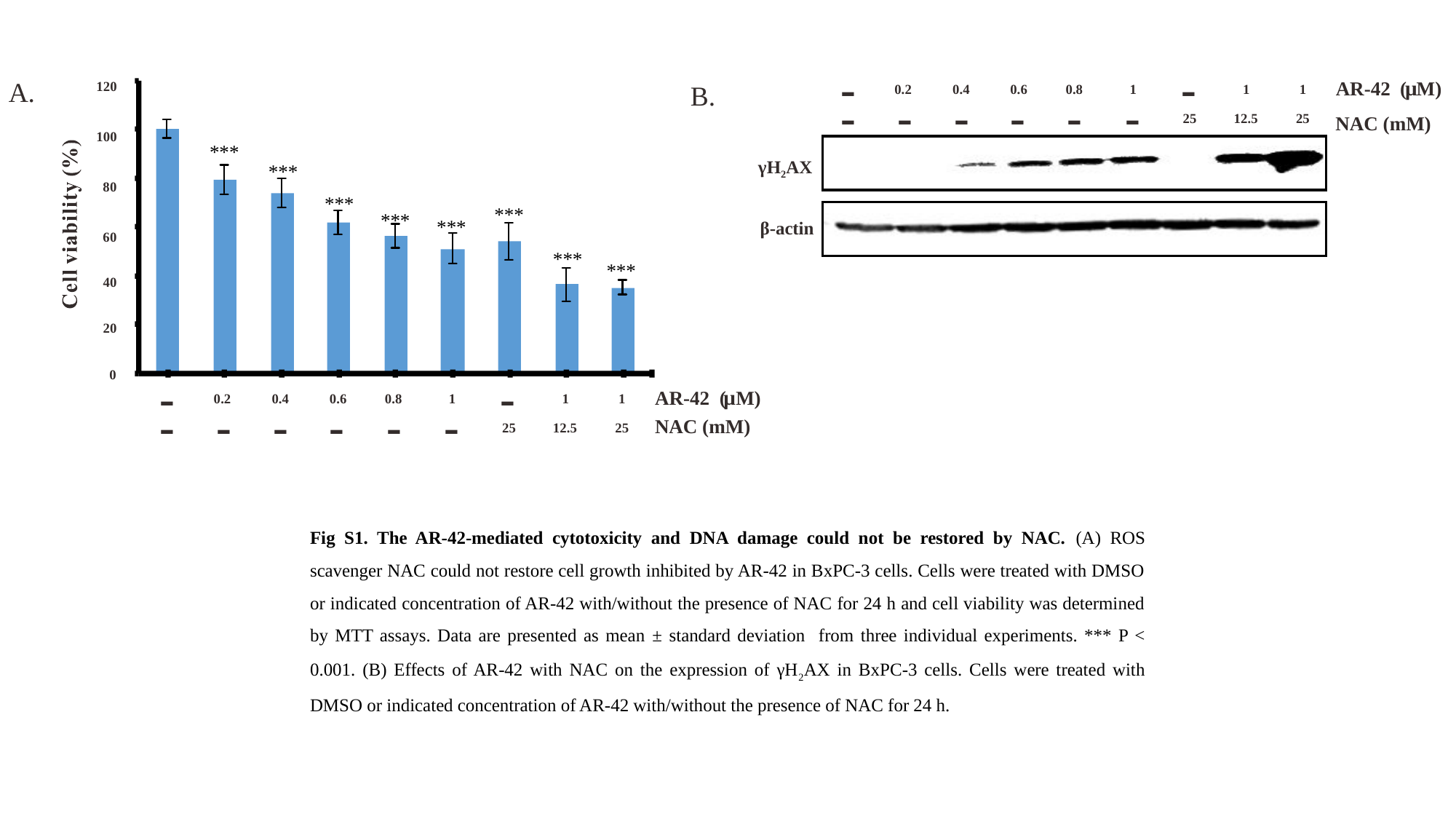

-
-
μ
A.
AR-42 ( M)
120
B.
0.2
0.4
0.6
0.8
1
1
1
-
-
-
-
-
-
25
12.5
25
NAC (mM)
100
***
γH2AX
***
80
***
***
***
***
β-actin
60
***
***
40
20
0
-
-
μ
AR-42 ( M)
0.2
0.4
0.6
0.8
1
1
1
-
-
-
-
-
-
NAC (mM)
25
12.5
25
Fig S1. The AR-42-mediated cytotoxicity and DNA damage could not be restored by NAC. (A) ROS scavenger NAC could not restore cell growth inhibited by AR-42 in BxPC-3 cells. Cells were treated with DMSO or indicated concentration of AR-42 with/without the presence of NAC for 24 h and cell viability was determined by MTT assays. Data are presented as mean ± standard deviation from three individual experiments. *** P < 0.001. (B) Effects of AR-42 with NAC on the expression of γH2AX in BxPC-3 cells. Cells were treated with DMSO or indicated concentration of AR-42 with/without the presence of NAC for 24 h.
