# Supplementary material for: Novel histone deacetylase inhibitor AR-42 exhibits antitumor activity in pancreatic cancer cells by affecting multiple biochemical pathways
Source: PLoS One. 2017 Aug 22;12(8):e0183368. doi: 10.1371/journal.pone.0183368 (PMC5567660; doi:10.1371/journal.pone.0183368)
Supplement: S3 Fig — (PPTX) [file pone.0183368.s006.pptx]

## Slide 1
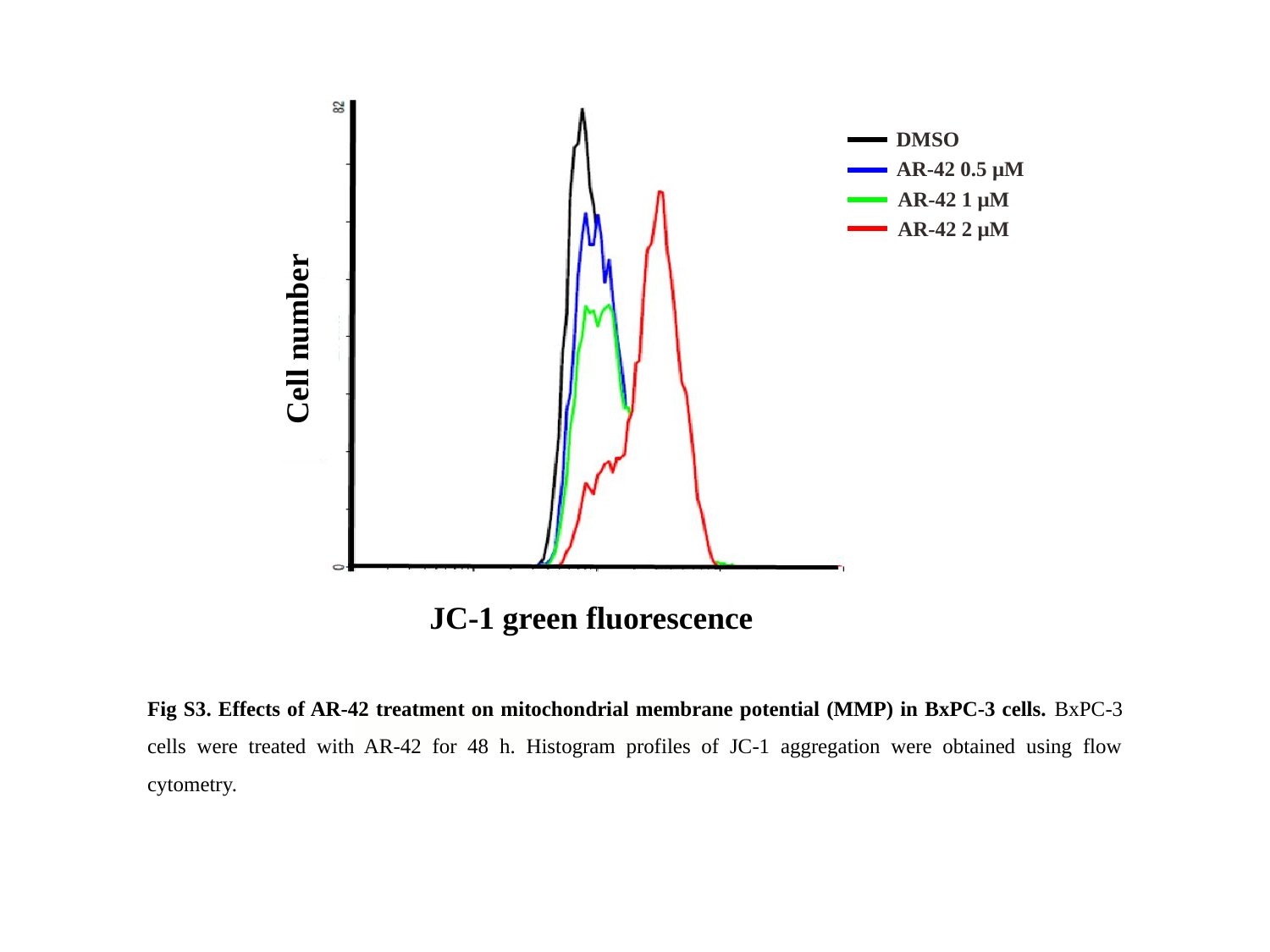

DMSO
AR-42 0.5 μM
AR-42 1 μM
AR-42 2 μM
Cell number
JC-1 green fluorescence
Fig S3. Effects of AR-42 treatment on mitochondrial membrane potential (MMP) in BxPC-3 cells. BxPC-3 cells were treated with AR-42 for 48 h. Histogram profiles of JC-1 aggregation were obtained using flow cytometry.
