# Supplementary material for: Novel histone deacetylase inhibitor AR-42 exhibits antitumor activity in pancreatic cancer cells by affecting multiple biochemical pathways
Source: PLoS One. 2017 Aug 22;12(8):e0183368. doi: 10.1371/journal.pone.0183368 (PMC5567660; doi:10.1371/journal.pone.0183368)
Supplement: S4 Fig — (PPTX) [file pone.0183368.s007.pptx]

## Slide 1
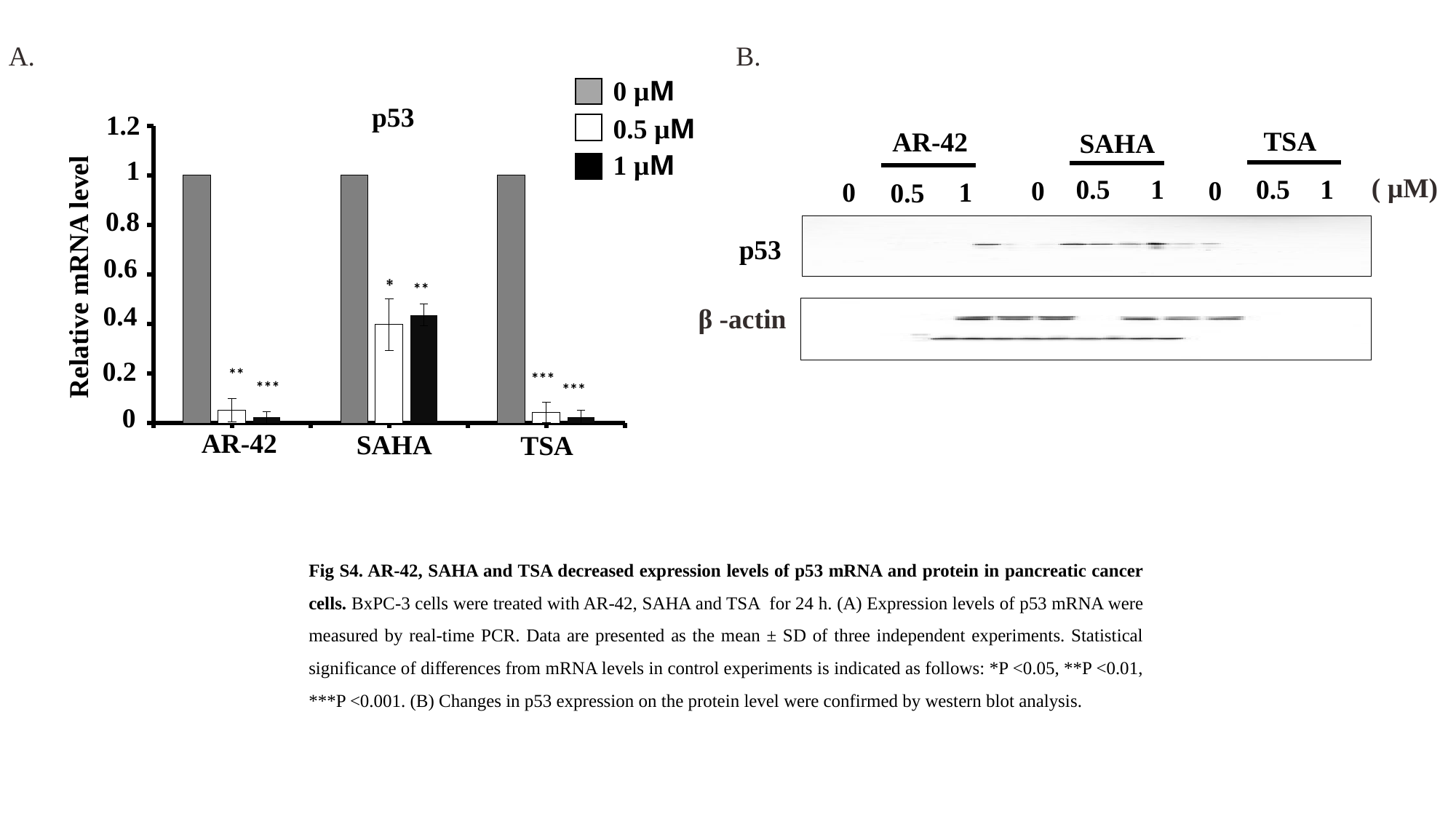

A.
B.
0 μM
p53
1.2
0.5 μM
TSA
AR-42
### Chart
| Category | AR-42 | SAHA | TSA |
|---|---|---|---|SAHA
1 μM
1
( μM)
1
0.5
0.5
1
0
0
1
0
0.5
0.8
p53
0.6
Relative mRNA level
0.4
β -actin
0.2
0
AR-42
SAHA
TSA
Fig S4. AR-42, SAHA and TSA decreased expression levels of p53 mRNA and protein in pancreatic cancer cells. BxPC-3 cells were treated with AR-42, SAHA and TSA for 24 h. (A) Expression levels of p53 mRNA were measured by real-time PCR. Data are presented as the mean ± SD of three independent experiments. Statistical significance of differences from mRNA levels in control experiments is indicated as follows: *P <0.05, **P <0.01, ***P <0.001. (B) Changes in p53 expression on the protein level were confirmed by western blot analysis.
