# Supplementary material for: Novel histone deacetylase inhibitor AR-42 exhibits antitumor activity in pancreatic cancer cells by affecting multiple biochemical pathways
Source: PLoS One. 2017 Aug 22;12(8):e0183368. doi: 10.1371/journal.pone.0183368 (PMC5567660; doi:10.1371/journal.pone.0183368)
Supplement: S5 Fig — (PPTX) [file pone.0183368.s008.pptx]

## Slide 1
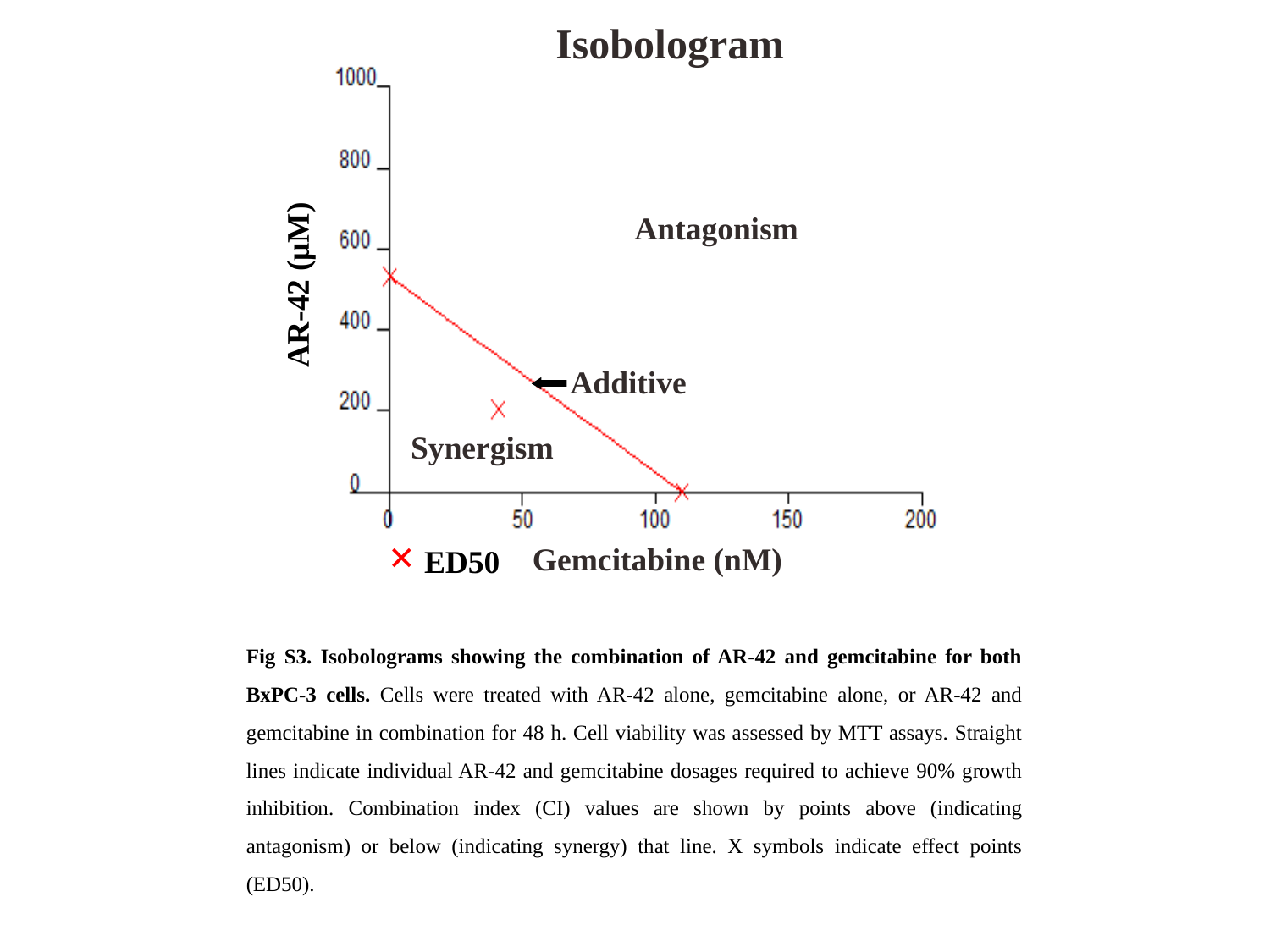

Isobologram
AR-42 (μM)
Antagonism
Additive
Synergism
× ED50
Gemcitabine (nM)
Fig S3. Isobolograms showing the combination of AR-42 and gemcitabine for both BxPC-3 cells. Cells were treated with AR-42 alone, gemcitabine alone, or AR-42 and gemcitabine in combination for 48 h. Cell viability was assessed by MTT assays. Straight lines indicate individual AR-42 and gemcitabine dosages required to achieve 90% growth inhibition. Combination index (CI) values are shown by points above (indicating antagonism) or below (indicating synergy) that line. X symbols indicate effect points (ED50).
